# Mitochondrial mRNA Processing in the Chlorophyte Alga *Pediastrum duplex* and Streptophyte Alga *Chara vulgaris* Reveals an Evolutionary Branch in Mitochondrial mRNA Processing

**DOI:** 10.3390/plants10030576

**Published:** 2021-03-18

**Authors:** Grayson C. R. Proulex, Marcus J. Meade, Kalina M. Manoylov, A. Bruce Cahoon

**Affiliations:** 1 Department of Natural Sciences, The University of Virginia’s College at Wise, 1 College Ave., Wise, VA 24293, USA; gcp5a@uvawise.edu (G.C.R.P.); mjm6mv@uvawise.edu (M.J.M.); 2 Department of Biological and Environmental Sciences, Georgia College and State University, Milledgeville, GA 31061, USA; kalina.manoylov@gcsu.edu

**Keywords:** mitochondria, RNA processing, algal evolution, circular RNA, polycytidylation, PacBio Iso-Seq

## Abstract

Mitochondria carry the remnant of an ancestral bacterial chromosome and express those genes with a system separate and distinct from the nucleus. Mitochondrial genes are transcribed as poly-cistronic primary transcripts which are post-transcriptionally processed to create individual translationally competent mRNAs. Algae post-transcriptional processing has only been explored in *Chlamydomonas reinhardtii* (Class: Chlorophyceae) and the mature mRNAs are different than higher plants, having no 5′ UnTranslated Regions (UTRs), much shorter and more variable 3′ UTRs and polycytidylated mature mRNAs. In this study, we analyzed transcript termini using circular RT-PCR and PacBio Iso-Seq to survey the 3′ and 5′ UTRs and termini for two green algae, *Pediastrum duplex* (Class: Chlorophyceae) and *Chara vulgaris* (Class: Charophyceae). This enabled the comparison of processing in the chlorophyte and charophyte clades of green algae to determine if the differences in mitochondrial mRNA processing pre-date the invasion of land by embryophytes. We report that the 5′ mRNA termini and non-template 3′ termini additions in *P. duplex* resemble those of *C. reinhardtii*, suggesting a conservation of mRNA processing among the chlorophyceae. We also report that *C. vulgaris* mRNA UTRs are much longer than chlorophytic examples, lack polycytidylation, and are polyadenylated similar to embryophytes. This demonstrates that some mitochondrial mRNA processing events diverged with the split between chlorophytic and streptophytic algae.

## 1. Introduction

Mitochondria are membrane-bound organelles known for supplying eukaryotic cells with energy through ATP to carry out cellular functions. This occurs due to aerobic respiration whereby pyruvate is oxidized to CO_2_ to generate reduced cofactors that drive the electron transport chain to chemiosmotically fuel ATP synthesis [1]. Despite the crucial role mitochondria have in supplying energy necessary for cellular functions and ATP for other biochemical pathways, it did not originate as a component of the eukaryotic cell. During the late 20th century, the theory of endosymbiosis became widely accepted and states that an aerobic bacterium was absorbed by, and formed an endosymbiotic relationship with, a pre-eukaryotic cell [2,3]. Though it became fully integrated into the Last Eukaryotic Common Ancestor (LECA), the proposed alpha-proteobacterium [4] maintained a portion of its circular genome carrying a conserved set of genes enabling the quick modulation of crucial energy acquisition proteins [5]. This remnant of the bacterial genome is referred to as mtDNA, the mitochondrial genome or chondriome.

Mitochondria have retained their own gene expression machinery, combining bacterial-like traits with novel features that evolved in the host cell [6]. Quite a bit is known about mitochondrial transcription and RNA processing from the compact chondriomes of humans and mice, which can serve for an overview of the process. Briefly, a nuclear-encoded RNA polymerase similar to those found in T3 and T7 bacteriophages [7] recognizes a promoter on both strands of mtDNA with the aid of two transcription factors [8,9]. These promoters occur in the only non-coding region (hyper-variable) and produce two long poly-cistronic primary RNAs known as heavy and light [10,11,12,13,14] with the aid of an elongation factor [14]. Individual mRNAs and tRNAs are removed from the primary transcripts by endonucleolytic cleavage by the enzymes RNaseP and RNaseZ, which precisely remove tRNAs, leaving most of the mRNAs as individual mRNAs with very short 3′ and 5′ UnTranslated Regions (UTRs), a process called the Punctuation Model [15,16,17]. Endonucleolytic processing between mRNAs with no intervening tRNA and between an mRNA with an adjacent antisense tRNA has been documented [13], but the enzymatic mechanisms responsible for these processing events are currently unknown. Having no 5′ UTRs, these mRNAs lack canonical ribosome binding sequences and use an alternative ribosome binding mechanism that is unique to mitochondria [18]. Once cleaved from the primary transcript, mRNAs may be polyadenylated, which adds the final adenine in some transcripts’ stop codons, stabilizes some, and acts as a degradation signal for truncated messages [19,20,21]. mRNA fragments, but not full-length mRNAs, may also be circularized [22].

The mitochondrial genomes of plants (embryophytes) are much larger than those in animal cells due to expansive intergenic regions, repetitive DNA, and introns [23]. Plant mitochondria share some transcriptional processes with vertebrates. Transcription in plant mitochondria is catalyzed by one or more nuclear encoded phage-like RNA polymerases [24,25], and transcription factors similar to those used by vertebrates are encoded in plant nuclear genomes [26], but their functions have yet to be demonstrated. Due to their sizes, plant chondriomes have multiple promoters dispersed throughout the chondriome [27], yielding multiple primary poly-cistronic transcripts. Post-transcriptional processing takes on an expanded role in plants requiring numerous RNA Processing Factors (RPFs) that target endo- and exo-nucleolytic enzymes, define mRNA ends, and modify transcripts [28]. The 5′ termini of genes directly downstream of a transcriptional promoter are formed by the initial nucleotide added by the RNA polymerase [29]. For downstream genes in poly-cistronic transcripts, endonucleolytic cleavage between two genes will simultaneously produce the 5′ UTR of one gene and the 3′ UTR of an adjacent one. The lengths of these UTRs range from dozens to thousands of nucleotides consistent with the large intergenic regions of plant chondriomes [29,30,31]. To date, the best-defined cleavage mechanisms in plants are the precise removal of tRNAs by RNaseZ and PRORP. Similarly, tRNA-like secondary structures called t-elements also define intergenic cleavage sites recognized by endonucleases [31,32,33,34,35]. Most protein-coding genes are not separated by tRNAs, and their intergenic cleavage mechanism is hypothetical at this time but involves at least two nucleases [30,36]. Multiple 5′ termini for each gene usually result from these processes [30,31]. The 3′ ends are less variable and gene specific RPFs bind to them, presumably defining and stabilizing them [37,38,39,40,41]. The prevalence of group I and II introns in plant mitochondria creates an added layer of post-transcriptional processing. Neither class of intron is able to self-splice, so a group of nuclear-encoded RPFs are necessary for their removal [42]. In addition to the major construction of the mRNA coding regions, individual nucleotides are modified in a process known as RNA editing, which is common in higher plant mitochondria and chloroplasts [43]. Once the mRNA is no longer needed, it may be marked for degradation by way of polyadenylation by nuclear encoded factors in a manner similar to that of bacteria [44,45,46,47].

Our understanding of mitochondrial transcription and RNA processing in algal species is mostly limited to the single-celled photosynthetic green alga *Chlamydomonas reinhardtii* P.A. Dangeard, which is a well-established model system [48]. *C. reinhardtii* has a small linear chondriome [49] that is unusual among algae but is a conserved trait among the Reinhardtinia clade of the Order Chlamydomonadales [50,51]. In this species, transcription is initiated on each of the two strands from promoters in a small intergenic region to produce two primary transcripts [52,53]. Each mRNA is endonucleolytically cleaved directly adjacent to the AUG start codon, leaving no 5′ UTR, similar to those seen in animal systems. The 3′ UTRs are comprised of various lengths of template-derived intergenic regions and may have non-template polycytosine and/or polyuracil tails added, presumably as part of the maturation process [54,55,56]. The poly-cytidylation of mitochondrial mRNAs seen in green algae is unusual and appears to be limited to the algal class Chlorophyceae [55]. It has been hypothesized that these leaderless mRNAs use an alternative ribosome-binding mechanism, but there is evidence that the mature mRNAs are circularized, which brings putative ribosome binding sites (RBSs) located in the intergenic regions of the *Chlamydomonas* chondriome upstream of the start codon to initiate translation [56]. mRNAs in *C. reinhardtii* are also poly-adenylated, which serves as a degradation signal consistent with mitochondria in other eukaryotes and bacteria [54,57,58]. mRNA editing, which is common among embryophytes, is missing in both the chlorophyte and streptophyte lineages of green algae [59], suggesting that some post-transcriptional processes were acquired by embryophytes after they invaded land.

The purpose of this study was to define the 5′ and 3′ UTRs of mitochondrial mRNAs in two algae, *Pediastrum duplex* Meyen (P: Chlorophyta, C: Chlorophyceae, F: Hydrodictyaceae) from the chlorophyte algal clade and *Chara vulgaris* Linnaeus (P: Charophyta, C: Charophyceae, F: Characeae) of the charophyte algal clade. *P. duplex* is a member of the same class as *C. reinhardtii*, but it has a circular chondriome that is several times larger [60,61], making the architecture more similar to that found in other algae. By defining the termini, we hoped to determine if RNA processing events seen in *C. reinhardtii* are also used among other chlorophytic algae or are related to its compact genome. *C. vulgaris* has a mitochondrial genome similar in size to *P. duplex*, but the gene content and synteny are more similar to bryophytes [62,63]. We analyzed the 3′ and 5′ ends from *C. vulgaris* to see if mRNA end processing resembled higher plants or chlorophytic green algae. Circular RT-PCR (cRT-PCR) and PacBio long-read sequencing were used to define the transcript termini of 12 mitochondrial mRNAs from each species. cRT-PCR allows the mapping of both the 3′ and 5′ ends of an RNA by artificially ligating them together followed by the production of a cDNA across the ligation site, PCR amplification of the sequences flanking the ligation site and sequencing of those amplicons. PacBio Iso-Seq is a long-read RNA sequencing technology which can sequence full-length RNAs including their 3′ and 5′ termini. This platform sequences poly-adenylated RNAs so organellar mRNAs must be artificially poly-adenylated to increase the likelihood they will be sequenced.

## 2. Results

### 2.1. Pediastrum duplex

**Figure 1:**
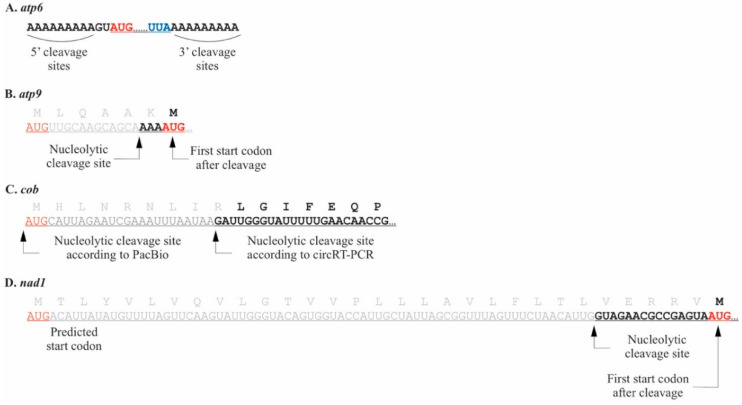
The 5′ termini and UTRs of four *P. duplex* mitochondrial mRNAs. Start codons are red, stop codons blue, and truncated portions of coding regions grey. (**A**). The 5′ UTR of the *atp6* mRNA occurred within a 9 nt templated stretch of adenines that also appears downstream of the stop codon. (**B**). The *atp9* 5′ terminus occurred downstream of the AUG start codon in archived *P. duplex* chondriomes, suggesting the protein may be six amino acids shorter than predicted. (**C**). Two *cob* 5′ termini were detected. Circular RT-PCR revealed one downstream of the AUG start codon in archived *P. duplex* chondriomes, while the PacBio Iso-Seq technique revealed a terminus directly adjacent to the predicted start codon. (**D**). The *nad1* 5′ terminus occurred downstream of the AUG start codon in archived *P. duplex* chondriomes, suggesting the protein may be thirty amino acids shorter than predicted.

In *P. duplex*, *cox2a*, *cox3*, *nad2*, *nad4*, and *nad4L* 5′ termini occurred directly upstream of the AUG start codon, essentially leaving no UTR (Table 1). For *atp6*, 5′ and 3′ end processing occurred within a 9 nt genomic DNA (gDNA) encoded stretch of adenines flanking the gene (Figure 1A). Since the same oligonucleotide sequence occurred at both ends, it was not possible to distinguish the exact location of the 5′ or 3′ exonucleolytic cleavage using cRT-PCR. Two of the *P. duplex* mRNAs (*atp9* and *nad1*) had cleavage sites producing 5′ UTR termini downstream of the predicted start codons in archived chondriome maps (KR026340, KR026340, MK895949). For *atp9* (Figure 1B) and *nad1* (Figure 1D), there is an in-frame AUG start codon adjacent to the cut site leaving a short 5′ UTR consistent with the other genes. For *cob*, there was disagreement in the 5′ terminus between the circRT-PCR and PacBio techniques. Using circ-RT-PCR, a single 5′ terminus 22 nt downstream of the predicted start codon was detected (Figure 1C), while reads using PacBio IsoSeq revealed a single terminus adjacent to the originally predicted start codon.

**Figure 2:**
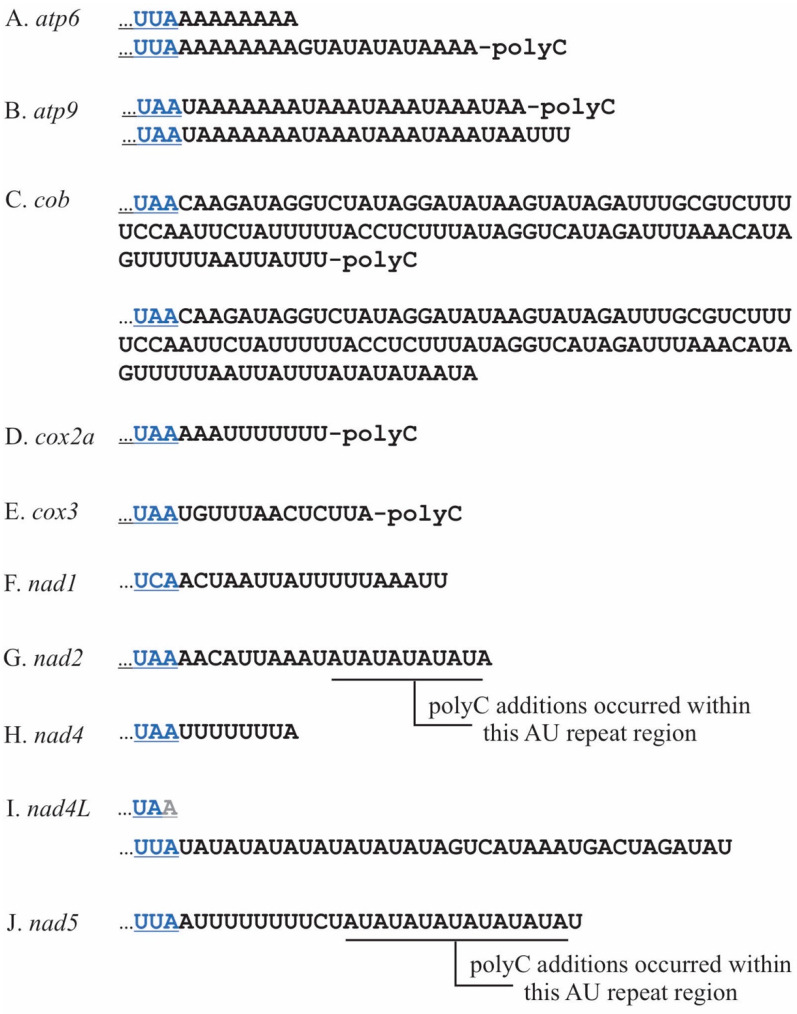
The 3′ termini and UTRs of ten *P. duplex* mitochondrial mRNAs. Stop codons are blue. (**A**). Two 3′ termini were detected for *atp6*. The upper (shorter) sequence had no oligonucleotide addition, while a portion of transcripts represented by the lower (longer) sequence had poly(C) additions. (**B**). For *atp9* a portion of the transcripts with the upper terminus had a poly(C) addition, while the slightly longer one represented by the lower sequence did not. (**C**). For *cob* a portion of the transcripts with the upper terminus had a poly(C) addition, while the one represented by the longer sequence did not. (**D**). For *cox2a* a single terminus was detected and a portion of them had a poly(C) tail. (**E**). A single terminus, some of which had a poly(C) addition, was detected for *cox3*. (**F**). A single terminus was detected for *nad1* and no oligonucleotide additions were detected. (**G**). For *nad2* poly(C) additions were detected at several different termini within an AU repeat region. (**H**). A single terminus was detected for *nad4* and no oligonucleotide additions were detected. (**I**). A single terminus was detected for *nad4L* and no oligonucleotide additions were detected. (**J**). For *nad5* poly(C) additions were detected at several different termini within an AU repeat region.

*P. duplex* 3′ UTR lengths were gene specific, with several having two or more termini (Figure 2A–J and Table 2). Most were relatively short, fewer than 25 nts, the exception being *cob* which were 100 and 110 nts in length (Figure 2C). Eight genes (*atp6*, *atp9*, *cob*, *cox1*, *cox2a*, *cox3*, *nad2*, and *nad5*) were polycytidylated. For some, this occurred at specific termini, e.g., *atp6*, *atp9*, *cob*, *cox1*, *cox2a*, and *cox3*. For two genes, *nad2* and *nad5*, the poly(C) additions occurred at variable locations within templated repetitive AU regions beginning 9nts downstream of the stop codons (Figure 2G,J and Table 2). No poly(C) additions were detected on *nad1*, *nad4*, or *nad4L*. On *nad4L*, there was an AU repeat region adjacent to the stop codon, but no poly(C) additions were detected. There was general agreement of the 3′ termini of fully processed mRNAs between cRT-PCR and PacBio reads for all but one transcript, *nad4L*. For this gene, cRT-PCR provided one 3′ terminus with a truncated stop codon (Figure 2J upper sequence), whereas PacBio data provided a 3′ terminus 37 nt downstream (Figure 2J lower sequence). The nucleotide sequences between the stop codon and the 3′ terminus for each gene were aligned and analyzed using a logo plot, and the 15 nucleotides upstream of the terminus were comprised nearly exclusively of adenines and uracils (Supplemental Figure S1A). These regions were analyzed for RNA secondary structure and none were detected.

**Figure 3:**
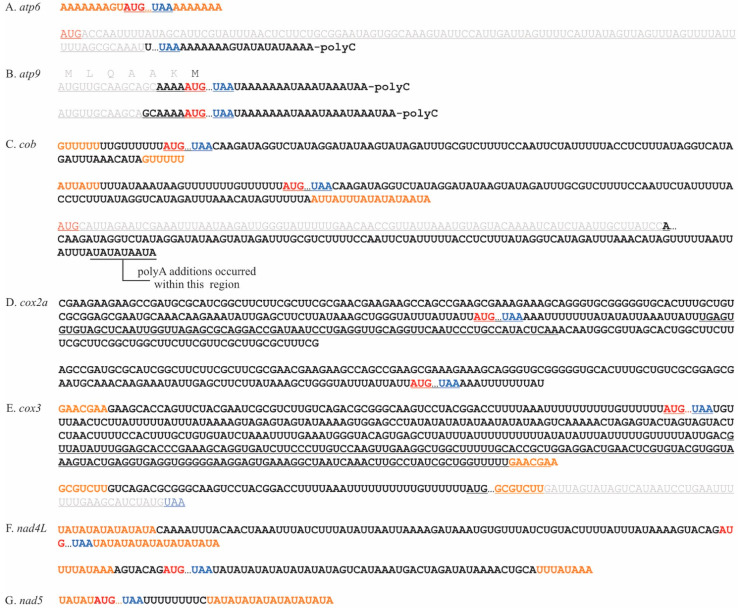
Naturally circularized mRNAs found in *P. duplex* mitochondria. Start codons are colored red, stop codons blue, repeat sequences orange, and truncated portions of coding regions grey. (**A**). Two circularized mRNAs were detected for the gene *atp6*, the upper sequence represents a full-length coding region whose 3′ and 5′ termini were ligated within templated adenine stretches that flank the coding region. The lower sequence represents a circularized portion of the mRNA. (**B**). Two versions of a circularized *atp9* transcript were detected. Both would be full length considering a start codon downstream of the previously predicted one (grey) would be the actual start codon. (**C**). Two full-length circularized versions of the *cob* transcript were detected (upper two sequences). The ligation termini coincided with two different repeat sequences (orange). A third circularized transcript with a truncated coding region was also detected for *cob*. (**D**). Two circularized full-length coding regions were detected for *cox2a*. (**E**). Two circularized *cox3* transcripts were detected, both ligations having occurred at repeat sequences. One carried a full-length coding region (upper) the other truncated (lower). (**F**). Two full-length circularized versions of the *nad4L* transcript were detected where ligation occurred at AU-rich repeat sequences. (**G**). One circularized *nad5* transcript was detected with the ligation occurring within AU-rich repeat sequences.

Naturally circularized mRNAs were also detected using RT-PCR. In *P. duplex*, circularized variants carrying full-length coding regions were detected for seven mRNAs (*atp6*, *atp9*, *cob*, *cox2a*, *cox3*, *nad4L*, and *nad5*) (Figure 3). For five of these, the circularization coincided with a tandemly repeated nucleotide motif. The ligation site for naturally circularized *atp6* transcripts occurred within a stretch of template coded adenines (Figure 3A). Several circularized variants of *cob* were found (Figure 3C), one where the circularization occurred within a template encoded polyU motif and a second within two AU rich motifs. Two *cox3* circular transcript variants were detected, one where the ligation occurred within a GAACGAA motif and a second ligated at a GCGTCTT motif that removed the final 45 nts of the coding region. Two *nad4L* circular variants were detected, each occurring within AT rich motifs and including full-length coding regions. Three naturally circularized transcripts were detected with poly(C) additions. The two circular variants of *atp6* and *cob* with a poly(C) addition had severely truncated coding regions (Figure 3A,C). Two *atp9* circular variants were detected and both had poly(C) additions and full-length coding regions (Figure 3B). A single circularized variant of the *cox2a* transcript was detected, with no obvious repeat motif and no poly(C) additions (Figure 3D).

**Figure 4:**
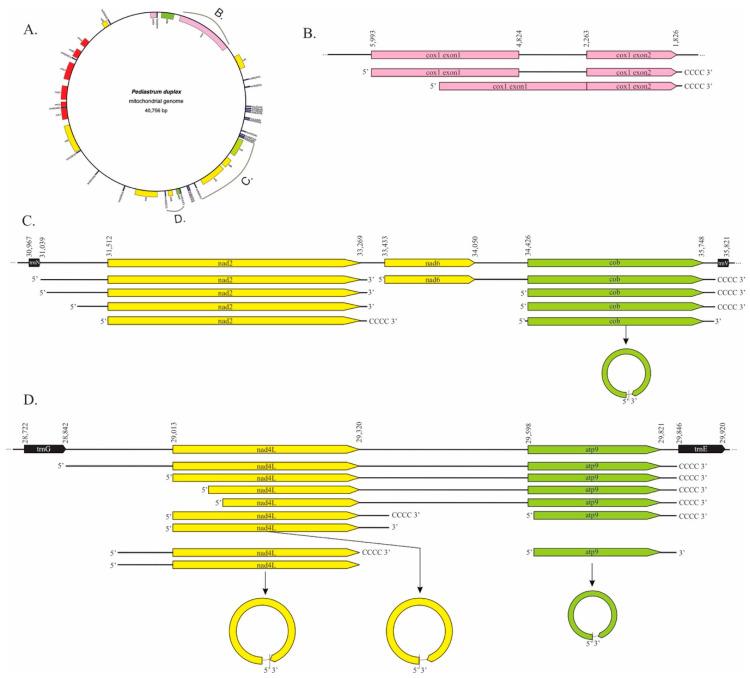
Mitochondrial RNA processing as determined by PacBio Iso-Seq and circRT-PCR. (**A**). The mitochondrial genome of *P. duplex* (GenBank MK895949). The regions highlighted in portions B, C, and D of this figure are marked. The map was generated using OGDRAW [64]. (**B**). The *cox1* gene, the upper diagram represents a portion of the chondriome, the lower two portions represent partially and fully processed transcripts. (**C**). The *trnG-nad2-nad6-cob-trnE* portion of the chondriome. (**D**). The *trnG-nad4L-atp9-trnE* portion of the chondriome.

The long-read PacBio data did not cover the entire chondriome or contain reads longer than ~2200 nt but, when combined with the circ-RT-PCR data, they did allow the detection of some broader transcript processing events from three portions of the *P. duplex* chondriome (Figure 4). For *cox1*, reads spanning the two exons and the intron were detected (Figure 4A). All of these reads had 5′ termini directly adjacent to the start AUG, while some were polycytidylated on the 3′ terminus. Transcripts with the intron removed were also detected that had the same 5′ and 3′ termini as the unspliced transcript. PacBio reads were also produced for another section with three genes, *nad2*-*nad6-cob*, flanked by tRNAs (Figure 4B). For *nad2*, transcripts appeared to have been endonucleolytically cleaved adjacent to *trnN*, forming a 5′ terminus for *nad2* and adjacent to *nad6* to form a 3′ terminus. The 5′ end was further processed, leaving multiple termini eventually resulting in an mRNA with no UTR that was also polycytidylated on the 3′ terminus. A transcript with both *nad6* and *cob* was detected. Its 5′ terminus occurred adjacent to *nad6′*s start codon, while the 3′ terminus appeared to have been created by the cleavage of *trnV* from the primary transcript followed by polycytidylation. The *cob* coding region was cleaved away from *nad6*, leaving two different 5′ termini. The linear transcripts were polycytidylated, whereas the circular version of *cob* was ligated with no poly(C) tract. No individual *nad6* transcript was detected from either circRT-PCR or PacBio results. A third region with the two genes *nad4L-atp9* flanked by tRNAs was also produced by the PacBio Iso-Seq methodology (Figure 4C). A single transcript that appeared to have been produced by the removal of the two tRNAs from the primary transcript was detected. The 3′ terminus of this poly-cistronic mRNA was poly-citidylated after *trnE* was removed. The removal of the *trnG* occurring 5′ of *nad4L* left 170 nt upstream of the start codon, but this was removed, leaving a 5′ terminus adjacent to the start AUG. The 3′ terminus was produced by endonucleolytic cleavage, leaving a 37 nt 3′ UTR that was polycytidylated. A second *nad4L* transcript was detected with a longer 5′ UTR (−80–94 nt within an AU repeat region) and a 3′ terminus comprised of a truncated stop codon that had been polycytidylated. Both versions of these shortest *nad4L* transcripts were detected as circular RNAs and neither contained a poly(C) tract. The *atp9* coding region was cleaved from the primary transcript, leaving a 5′ terminus −2 nt upstream of its start AUG, but its 3′ terminus was the one formed by the removal of *trnE*. A non-polycytidylated version of this mRNA was circularized.

Since tRNA removal was found to be integral in the maturation of *cob* and *atp9* 3′ termini, the placement of tRNAs was compared to the 3′ termini of other genes (Table 2). Five of the genes we analyzed had a 3′ adjacent tRNA, but only two, *cob* and *atp9*, had mature 3′ ends matching the placement of those tRNAs. The possibility of t-elements was considered for the other genes, but no evidence of secondary structures immediately downstream of the mature 3′ ends was detected for *atp6*, *cox1*, *cox2a*, *cox3*, *nad1*, *nad2*, *nad4*, *nad4L*, *nad5*, or *nad6*.

### 2.2. Chara vulgaris

**Figure 5:**
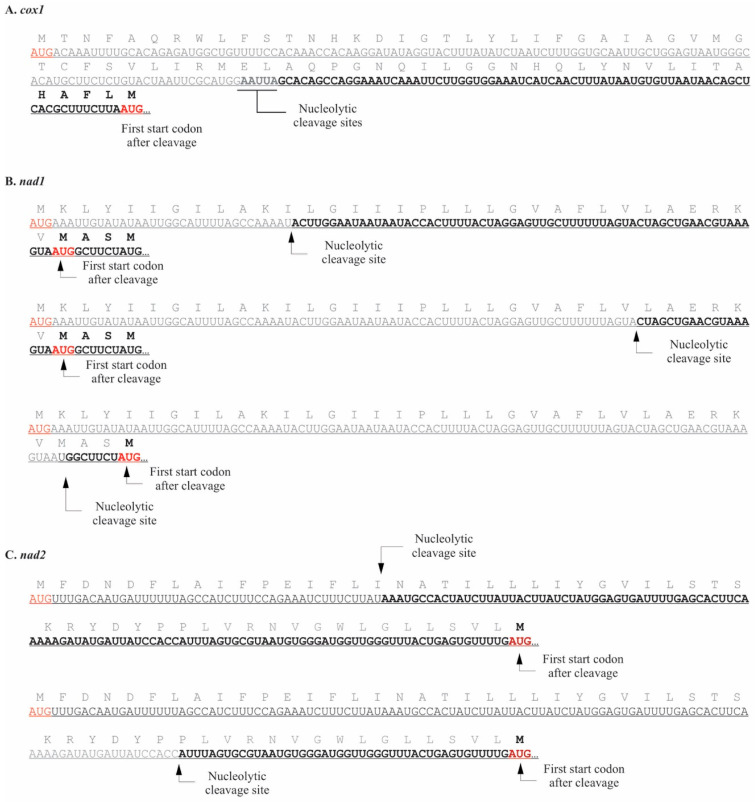
The 5′ termini and UTRs of three *C. vulgaris* mitochondrial mRNAs where the 5′ terminus occurred downstream of predicted start codons. Start codons are red and truncated portions of coding regions grey. (**A**). For *cox1* several 5′ termini were detected within a 5-nucleotide region. (**B**). Three 5′ termini were detected for *nad1*. (**C**). Two different termini were detected for *nad2*.

The 5′ UTRs were much longer than those observed in *P. duplex* (Table 3). Based on the termini we detected, UTRs ranged from 6–273 nucleotides, with an average length of 80 nts (S.E. = 15 nt). They were also more variable, with two or more termini detected for seven genes, *atp6*, *cob*, *cox2*, *cox3*, *nad1*, *nad4*, and *nad4L*. For three of the genes, *cox1*, *nad1*, and *nad2*, the mapped 5′ termini occurred downstream of the start codons in GenBank record NC_005255 (Figure 5). For *cox1*, a single terminus was detected 125–129 nts downstream of the predicted start codon. The next start AUG occurs 75–79 nt downstream (Figure 5A). Three 5′ UTRs were detected for *nad1*, all of which remove the predicted start codon but, depending upon the cleavage site, leave two possible alternative AUG start codons (Figure 5B). In *nad2*, two 5′ UTRs were detected, both of which leave a single alternative AUG start codon (Figure 5C). The length of the 5′ UTRs in *C. vulgaris* raised the possibility that they may fold to form RNA secondary structures, but the probability of secondary structures in the 5′ UTRs was found to be extremely low. The coverage of chondriome derived transcripts for *C. vulgaris* using PacBio sequencing was very low, so the 5′ termini of only two genes was recovered, *atp9* and *cox2*. Neither were the same length as those detected by circRT-PCR (Table 3).

**Figure 6:**
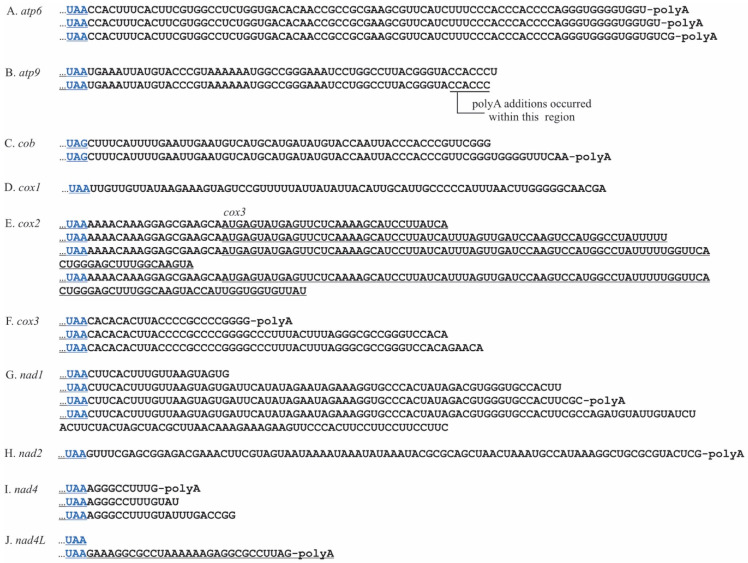
The 3′ termini and UTRs of ten *C. vulgaris* mitochondrial mRNAs. Stop codons are blue. (**A**). Three termini were detected for *atp6*, a portion of each had a poly(A) addition. (**B**). Several termini were detected for *atp9*. The longer one (upper) had no oligonucleotide additions, while those lacking the terminal uracil had poly(A) additions within a 6 nt region. (**C**). Two *cob* termini were detected and the shorter one had no detectable oligonucleotide additions while the longer (lower) one did. (**D**). A single terminus was detected for *cob* with no oligonucleotide additions. (**E**). Four termini were detected for *cox2*. All had a portion of the adjacent *cox3* gene and no oligonucleotide additions. (**F**). Three *cox3* termini were detected. A portion of the shortest UTR had poly(A) tails, while the two longer ones did not. (**G**). Four termini were detected for *nad1* but only one had a poly(A) tail. (**H**). A single terminus was detected for *nad2* and a portion of them had a poly(A) tail. (**I**). Three termini were detected for *nad4* and a portion of the shortest had a poly(A) tail. (**J**). Two termini were detected for *nad4L*. One occurred directly adjacent to the stop codon. A portion of the longer UTR had a poly(A) addition.

*C. vulgaris* 3′ ends ranged from 0–162 nts with an average of 61.3, S.E. = 7.5, (Table 4, Figure 6). Multiple 3′ termini were detected for each gene, except *cox1*, and *nad2*, which had single termini. Polyadenylation was detected on eight genes (*atp6*, *atp9*, *cob*, *cox3*, *nad1*, *nad2*, *nad4*, and *nad4L*). For genes where multiple termini were detected, the polyA tail only occurred on one of those termini (Figure 6B,C,F,G,I). The exception was *atp6*, where all three termini were polyadenylated (Figure 6A). The proportion of those specific transcripts with polyA additions varied considerably. For example, 0.7% of *atp9* transcripts with 52–57 nt 3′ UTRs had a polyA tail, whereas the majority of specific *nad2*, *nad4*, and *nad4L* transcripts were tailed. PacBio sequencing produced data for four genes and the 3′ termini agreed with three (Table 4). The exception was *cob*, where the PacBio sequencing revealed a longer 3′ UTR than those found with circRT-PCR. PacBio sequencing could not be used to detect non-template poly(A) tails since the mitochondrial transcripts were artificially polyadenylated to accommodate the Iso-Seq technique. The forty nucleotides upstream of the 3′ termini were analyzed for conserved sequences using a logo plot and none were found (Supplemental Figure S1B). The length of the 3′ UTRs in *C. vulgaris* raised the possibility that these regions could fold into secondary structures. Secondary structure prediction suggested a high probability that stable stem-loop structures occur adjacent to the 3′ terminus in all but one (*cox2*) of these 3′ UTRs (Supplemental Figure S2).

Since tRNA placement was found to be important for 3′ maturation of some *P. duplex* genes, the distances of mature 3′ termini from the stop codons of genes with an adjacent tRNA were compared for *C. vulgaris* (Table 4). For the twelve genes used in this study, none had 3′ termini formed from the removal of an adjacent tRNA. The presence of RNA secondary structure (t-elements) immediately downstream of 3′ termini was also checked, and three genes (*atp6*, *nad1*, and *nad2*) had potential stem-loop structures adjacent to their mature 3′ termini.

**Figure 7:**
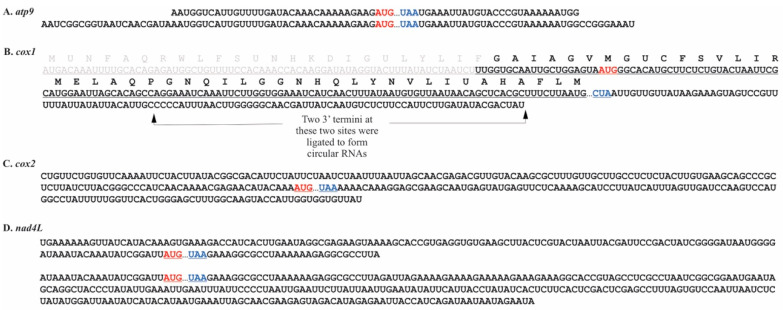
Naturally circularized mRNAs found in *C. vulgaris* mitochondria. Start codons are colored red, stop codons blue, and truncated portions of coding regions grey. (**A**). Two circularized versions of full-length transcripts were detected for *atp9*. (**B**). Two circularized *cox1* transcripts were detected. Both had the same 5′ end but different termini on the 3′ ends. (**C**). A single circularized version of the *cox2a* transcript was detected. (**D**). Two circularized full-length *nad4L* transcripts of different lengths were detected.

In *C. vulgaris*, circularized full length coding regions were detected for five genes. Circularized *nad2* transcripts matched the 3′ and 5′ termini detected in earlier experiments (Figure 5C and Figure 6H), suggesting that only naturally circularized transcripts were detected for this gene. For the genes *atp9*, *cox1*, *cox2*, and *nad4L*, circularized variants differed from the artificially circularized transcripts analyzed in previous experiments and are represented in Figure 7. For genes *cox2*, *cox3*, *nad1*, and *nad4*, only fragments of coding regions were found circularized. In *C. vulgaris*, there were no repeat motifs associated with the ligation sites and no polyA additions detected in the circularized transcripts.

## 3. Discussion

Green algae form two discrete clades, chlorophytes and charophytes [65]. Chlorophytes are morphologically diverse, cosmopolitan and contain the majority of green algae. Chlorophyte chondriomes are nearly as diverse as their morphologies, ranging from 15,500–66,000 bp with few introns and highly variable intergenic spaces, gene content, and gene synteny [60,66,67,68,69,70,71,72,73,74,75]. The knowledge of transcription and post-transcriptional processing events in chlorophytic mitochondria is limited to the model system *C. reinhardtii*, where mature mRNAs have no 5′ UTR and relatively short 3′ termini that may be polycytidylated. *C. reinhardtii* has a very small linear chondriome that is not representative of the majority of algae, and it is unknown if the post-transcriptional processing characteristics are hallmarks of its reduced chondriome or traits conserved among chlorophytes. Charophytes diverged from chlorophytes a billion or more years ago and have biochemical and morphological synapomorphies shared only with embryophytes [76]. The relatively few extant species belong to six monophyletic clades and are the closest living algal relatives of land plants [63,77]. The chondriomes of charophytes range widely from 56,500 bp to >201,000 bp, with highly variable gene order, density, and intron placement [63].

### 3.1. Mitochondrial Processing in P. duplex Resembles That Seen in C. reinhardtii

We mapped the 5′ and 3′ termini of presumably mature *P. duplex* mitochondrial mRNAs and found the 5′ UTRs to be very short or non-existent, the 3′ UTRs of varying lengths, the polycytidylation of 3′ termini, and circularized full-length mRNAs. In the only other chlorophyte where mitochondrial RNA processing has been documented, *C. reinhardtii*, individual mRNAs are endonucleolytically cleaved from primary transcripts directly adjacent to the start codons, leaving no 5′ UTR. The remaining intergenic region becomes the 3′ UTR of an adjacent gene and is of varying lengths, presumably due to exonucleolytic processing [56]. It has also been demonstrated that non-template polycytidylation occurs on the 3′ termini of *C. reinhardtii*, and that this phenomenon may be limited to the Phylum Chlorophyta since it has been found in representative species from Peridinophyceae, Prasinophyceae, Trebouxiophyceae, and Chlorophyceae, but not in a red alga (*Chondrus crispus* Stackhouse), a glaucophyte (*Cyanophora paradoxa* Korshikov), or embryophytes (*Physcomitrella patens* (Hedw.) Bruch & Schimp., *Arabidopsis thaliana* (L.) Heynh., and *Solanum tuberosum* L.) [55]. *Chlamydomonas* mitochondrial mRNAs are also 3′ polyuridylylated and polyadenylated [54,55,56,57], but this was not observed in *P. duplex*. 

Circular mRNAs are a common phenomenon across the biological spectrum, but their purpose has been difficult to determine [78]. In algal mitochondria mRNA circularization has been demonstrated in *Chlamydomonas*, where it appears to create translatable mRNAs and was hypothesized to be linked to polycytidylation [56]. In *P. duplex*, full-length coding regions were found to be circularized for seven of the twelve transcripts that were analyzed. The remaining five yielded no data so we are uncertain if they form circular transcripts. Circularization for five of the transcripts coincided with tandemly repeated template-derived motifs, AU repeats, GAACGAA, and GCGUCUU, which was not reported in *C. reinhardtii*. RNA circularization among nuclear genes occurs by way of an intron-exon back-splicing mechanism involving protein factors and conserved cis-elements [79,80], and perhaps a similar mechanism involving repeated elements occurs in mitochondria. The co-incidence of poly-cytidylation and circularization in *C. reinhardtii* led to a hypothesis that the poly(C) acted as a cis-element for circularization. In *P. duplex*, only one transcript, *atp9*, had circular mRNAs with full-length coding regions and a poly(C) addition. The other two transcripts with poly(C) additions, *atp6* and *cob*, were fragments of the coding region, suggesting that the poly(C) addition is not universally linked to the creation of translatable circular mRNAs and may not act as a signal for circularization. 

These data demonstrate that mitochondrial mRNA processing is conserved in the Chlorophyceae algal clade. The polycytidylation of mitochondrial mRNAs has already been shown to be conserved across the chlorophytic algae [55]. This study extends the similarities of mitochondrial mRNA processing to include the absence of a 5′ UTR, despite *P. duplex* having larger intergenic regions than *C. reinhardtii*. Our data suggest that the cleavage of tRNAs plays a pivotal role in the maturation of two mRNAs (*cob* and *atp9*) in *P. duplex*, which is consistent with mitochondrial transcripts in all systems studied to date, from *C. reinhardtii* to humans. The difference is the size of the chondriome and the lengths of the intergenic regions. We found that the removal of tRNAs in *P. duplex* creates mature 3′ ends that, at least in two cases, require no further processing other than polycytidylation. The 5′ ends, however, are further processed until no 5′ UTR remains (Figure 4). The lack of a 5′ UTR and the accompanying translation mechanism required is not only due to the compressed chondriome of *C. reinhardtii*, but also its purposeful removal prior to translation. *Pediastrum* is in the order Sphaeropleales and is a sister clade to Volvocales, which contains *Chlamydomonas* [81], so it is possible that these processing events could be limited to those two Orders; however, we hypothesize that these processes are conserved across the Chlorophyceae.

### 3.2. Mitochondrial Processing in C. vulgaris Resembles That Seen in Embryophytes.

We also analyzed the mRNA termini of the mitochondrial mRNAs of *C. vulgaris* and found the 5′ and 3′ UTRs to be much longer and more variable than those observed in *P. duplex* and *C. reinhardtii*. We also detected non-template polyadenylation of 3′ termini, the possibility of RNA secondary structures in processed 3′ UTRs, and the possibility of t-elements downstream of some 3′ termini. The *C. vulgaris* chondriome is one of the smaller among charophytic algae, 67,737 bp, with intergenic region sizes more similar to Chlorophyeae than higher plants, yet with gene content, gene synteny, and intron placement very similar to the much larger bryophyte chondriomes [62,63]. After post-transcriptional processing, we found that the 3′ and 5′ UTR lengths in *C. vulgaris* were more similar to those found in embryophytes than chlorophytes. In embryophytes, 5′ termini are of varying lengths, ranging from dozens to thousands of nucleotides in length, presumably due to a stepwise 5′ maturation process [29,30,31,32,33,34,35,36,82,83,84,85,86]. Among embryophytes, 3′ UTR lengths tend to be more consistent in length and mature mRNAs lack non-template oligonucleotide additions other than polyadenylation, which marks the mRNA for degradation [44,45,46,47]. Embryophyte mitochondria utilize tRNAs and RNA secondary structure (t-elements) to guide endonucleolytic cleavage [31,32,33]. The presence of relatively long 5′ and 3′ UTRs, the polyadenylation of some 3′ termini, the possibility that secondary structures may guide the formation of some 3′ termini, and the possibility that secondary structures may form in the 3′ UTRs of *C. vulgaris* mitochondrial mRNAs to presumably provide a target for 3′ specific RPFs demonstrates that their RNA processing is more similar to embryophytes than chlorophytes. One recent proteomic survey of *Arabidopsis* mitochondria estimated that 14.9% of the nuclear encoded mitochondrial-targeted proteome is devoted to mRNA processing [87]. Our results suggest that the RNA processing mechanisms and perhaps the large numbers of nuclear-encoded RPFs that locate to the mitochondria in higher plants were present in the common ancestor of all streptophytes.

In *C. vulgaris*, circularized full length coding regions were detected for five genes and only fragments were found for four other genes. Unlike *P. duplex*, there were no repeat motifs associated with the ligation sites and no polyA additions detected in the circularized transcripts. Among embryophyte mitochondria, circularized RNAs were characterized in *Hordeum vulgare* and *Arabidopsis*, but all of the examples were fragmented coding regions [88,89]. Our data suggest that the circularization of mRNAs in *C. vulgaris* may be a hybrid of chlorophytes and embryophytes.

## 4. Materials and Methods

### 4.1. Cultures

The *P. duplex* strain used in this study was morphologically identified and its chondriome sequenced and archived in GenBank (MK895949), as described in [58]. It has been maintained in Bold’s basal medium [90] in an Erlenmeyer flask on a lab bench with ambient light (adjacent to a window). The *C. vulgaris* strain was collected from a pond on the University of Virginia’s College at Wise campus in 2015 and cultured in an aquarium containing mud substrate collected from the same pond. This culture has been maintained in a greenhouse and supplemented with commercial plant fertilizer [59]. Its chondriome has been fully sequenced and is nearly identical to the one archived by Turmel et al. [62] (NC-005255).

### 4.2. RNA Extraction and CircRT-PCR

To promote RNA production, *P. duplex* cultures were incubated in a shaking incubator (50 rpm) at constant temperature (28 °C) and artificial light (500 μE m^−2^ s^−1^) for several hours prior to extraction. Cells were pelleted using a Beckman-Coulter Avanti JXN-30 centrifuge, and pellets were resuspended in Qiagen’s RNeasy extraction buffer (Germantown, MD, USA) and transferred to a bead beater tube. Cells were lysed by bead beating on a vortexer for five minutes. The lysate was then taken through the remaining Qiagen RNeasy protocol with the optional DNase step. *C. vulgaris* tissues were flash frozen in liquid nitrogen and ground with a mortar and pestle followed by RNA extraction using a Qiagen RNeasy kit, as described in Cahoon et al. [59]. All RNA samples were quantified using a NanoDrop Lite (Thermo-Fisher, Waltham, MA, USA) and stored at −80 °C. 

Primers for cDNA synthesis and PCR (Supplemental Table S1) were designed using Primer3 (https://primer3.org) and synthesized by Integrated DNA Technologies (Coralville, IA, USA). RT-PCR was completed as described in Meade et al. [91]. Briefly, RNAs were artificially circularized using 2 μg of total RNA and T4 RNA ligase (New England Biolabs, Ipswich, MA, USA). cDNAs were synthesized from the circularized RNAs using the R1 primers and MMLV Reverse Transcriptase (Promega, Madison, WI, USA). These cDNAs were then used as template, along with the R1 and L1 primers to PCR amplify the 3′–5′ junctions of each transcript using Phusion DNA polymerase (ThermoFisher, Waltham, MA, USA). The products of these PCR reactions were diluted 10-fold and used as template for a second round of PCR using primers R2 and L2. This process was completed twice with RNA extracted at two different times to represent independent replicates. Naturally circularized mRNAs were detected producing cDNA directly from 2 μg of total RNA without T4 ligase treatment followed by two rounds of PCR as described above. The naturally circularized mRNA process was also completed twice.

### 4.3. Sequencing and Analysis

The independent replicates of the secondary PCR products for both *P. duplex and C. vulgaris* were deep sequenced, separately, using Genewiz’s Amplicon EZ Illumina MiSeq service (South Plainfield, NJ, USA). Sequences were analyzed using Geneious Prime software (Biomatters, Auckland, New Zealand). Initially, sequences were matched to each gene using the Map-to-Reference function. These sequences were visually inspected, for 3′ poly-nucleotide additions, and sequence motifs. The 3′–5′ junction sites were identified using NCBI’s BLAST align two or more sequences feature (https://blast.ncbi.nlm.nih.gov/Blast.cgi). 

For PacBio Iso-Seq, ~1 g of total RNA from each species was polyadenylated using Lucigen’s (Middleton, WI, USA) Poly(A) Polymerase Tailing kit according to the manufacturer’s protocol. The tailed samples were cleaned using Qiagen’s RNeasy kit. Samples were shipped to GeneWiz on dry ice for PacBio library preparation and sequencing. This process was completed once. Reads were aligned to the *P. duplex* and *C. vulgaris* chondriomes with Geneious Prime software using the Map-to-Reference function. 

Logo plots were generated using the web-based service https://weblogo.berkeley.edu/logo.cgi. RNA secondary structures were predicted using RNAfold (http://rna.tbi.univie.ac.at//cgi-bin/RNAWebSuite/RNAfold.cgi).

## 5. Conclusions

We present evidence of mitochondrial mRNA processing from two green algae from the chlorophyte and streptophyte lineages with similarly sized circular mitochondrial genomes. The primary differences were the absence of secondary structures (t-elements) in the *P. duplex* chondriome that are important for processing in streptophytes, and the removal of the 5′ UTR in chlorophytes but not streptophytes. We hypothesize that t-elements were gained and the 5′ UTR processing lost in the common ancestor of the streptophytic algae. We also confirm the polycytidylation of the 3′ termini of *P. duplex* but not *C. vulgaris*, which is consistent with the theory that poly(C) addition is limited to chlorophytes.

## Figures and Tables

**Table 1:** Site of 5′ UTR terminus upstream from start codon (in nucleotides) in *Pediastrum duplex* mitochondria, nd = no data. * Some 5′ UTR termini detected in this study occur downstream of the start codons in archived chondriomes. The distances presented in this table are marked from the next available AUG start codon.

	Site of 5′ UTR Terminus Upstream from Start Codon (in Nucleotides)	
Gene	Circular RT-PCR	PacBio
*atp6*	−2–11	nd
*atp9*	−3 *	nd
*cob*	−2 *	0
*cox1*	nd	0
*cox2a*	0	nd
*cox3*	0	0
*nad1*	−15 *	nd
*nad2*	0	nd
*nad4*	0	0
*nad4L*	0	0
	−81–93	nd
*nad5*	nd	nd
*nad6*	nd	0

**Table 2:** Lengths of the most abundant 3′ UTRs found in *Pediastrum duplex* and those found with a non-template addition

	Circular RT-PCR				PacBio Long Reads		
Gene	Length of 3′ UTR from Stop Codon	UTRs with polyC Addition	UTRs with polyA + polyC Addition	UTR with polyA Addition	Length of 3′ UTR from Stop Codon	UTRs with polyC Addition	Genes with an Adjacent tRNA (Distance from Stop Codon in nt)
*atp6*	21	+	-	-	nd	nd	
	0–9	-	-	-	nd	nd	
*atp9*	22	+	-	-	16–22	+	26
	25	-	-	-	nd	nd	
*cob*	110	-	-	-	104–110	+	110
	100	+	-	-	nd	nd	
*cox1*	13	+	-	-	7–14	+	
*cox2a*	10	+	-	-	nd	nd	28
*cox3*	13	+	-	-	10–13	-	
*nad1*	18	-	-	-	nd	nd	
*nad2*	9–21	+	-	-	5–23	-	
*nad4*	8	-	-	-	7–8	+	786
*nad4L*	−1	-	-	-	37	-	
*nad5*	0–8	-	-	+	1–3	-	42
	9–24	+	+				
*nad6*	nd	nd	nd	nd	nd	nd	

nd: no data.

**Table 3:** Site of 5′ UTR terminus upstream from start codon (in nucleotides) in *Chara vulgaris* mitochondria, nd = no data. * 5′ UTR termini detected in this study occur downstream of the start codons found in archived chondriomes. The distances presented in this table are marked from the next available AUG start codon.

	Site of 5′ UTR Terminus Upstream from Start Codon (in Nucleotides)	
Gene	Circular RT-PCR	PacBio
*atp6*	−76	nd
	−62	
*atp9*	−56	−93
*cob*	−161	nd
	−46	
*cox1*	−150 *	nd
*cox2*	−318	−52
	−18	
*cox3*	−153	nd
	−31	
*nad1*	−73 *	nd
	−27 *	
	−8 *	
*nad2*	−112 *	nd
*nad4*	−273	nd
	−107	
	−99	
	−82	
	−18	
	−6	
*nad4L*	−260	nd
	−18	
*nad5*	nd	nd
*nad6*	−36	nd

**Table 4:** Length of the most abundant 3′ UTRs found in *Chara vulgaris* and those with a non-template polynucleotide addition.

	Circular RT-PCR		PacBio		
Gene	Length of 3′ UTR from Stop Codon	UTRs with polyA Addition	Length of 3′ UTR from Stop Codon	Genes with an Adjacent tRNA (Distance from Stop Codon in nt)	Evidence of RNA Secondary Structure Immediately Downstream of 3′ Terminus
*atp6*	79	+	nd	459	+
	81	+			
	83	+			
*atp9*	52–57	+	56	92	-
	58	-			
*cob*	54	-	121		-
	68	+			
*cox1*	73	-	73		
*cox2*	51	-	nd		-
	82	-			
	107	-			
	162	-			
*cox3*	23	+	52	6056	-
	51	-			
	56	-			
*nad1*	20	-	nd		+
	67	-			
	70	+			
	141	-			
*nad2*	87	+	nd	1189	+
*nad4*	10	+	nd		-
	13	-			
	21	-			
*nad4L*	0	-	nd	2508	-
	29	+			
*nad5*	nd	nd	nd		
*nad6*	nd	nd	nd	5	

nd: no data.

## Supplementary Materials

The following are available online at https://www.mdpi.com/2223-7747/10/3/576/s1, Figure S1: Logo plots, Figure S2: Hypothetical RNA secondary structures, Table S1: DNA oligo primers.
