# Supplementary figures and images for: Mitochondrial mRNA Processing in the Chlorophyte Alga Pediastrum duplex and Streptophyte Alga Chara vulgaris Reveals an Evolutionary Branch in Mitochondrial mRNA Processing
Source: Plants (Basel). 2021 Mar 18;10(3):576. doi: 10.3390/plants10030576 (PMC8003010; doi:10.3390/plants10030576)

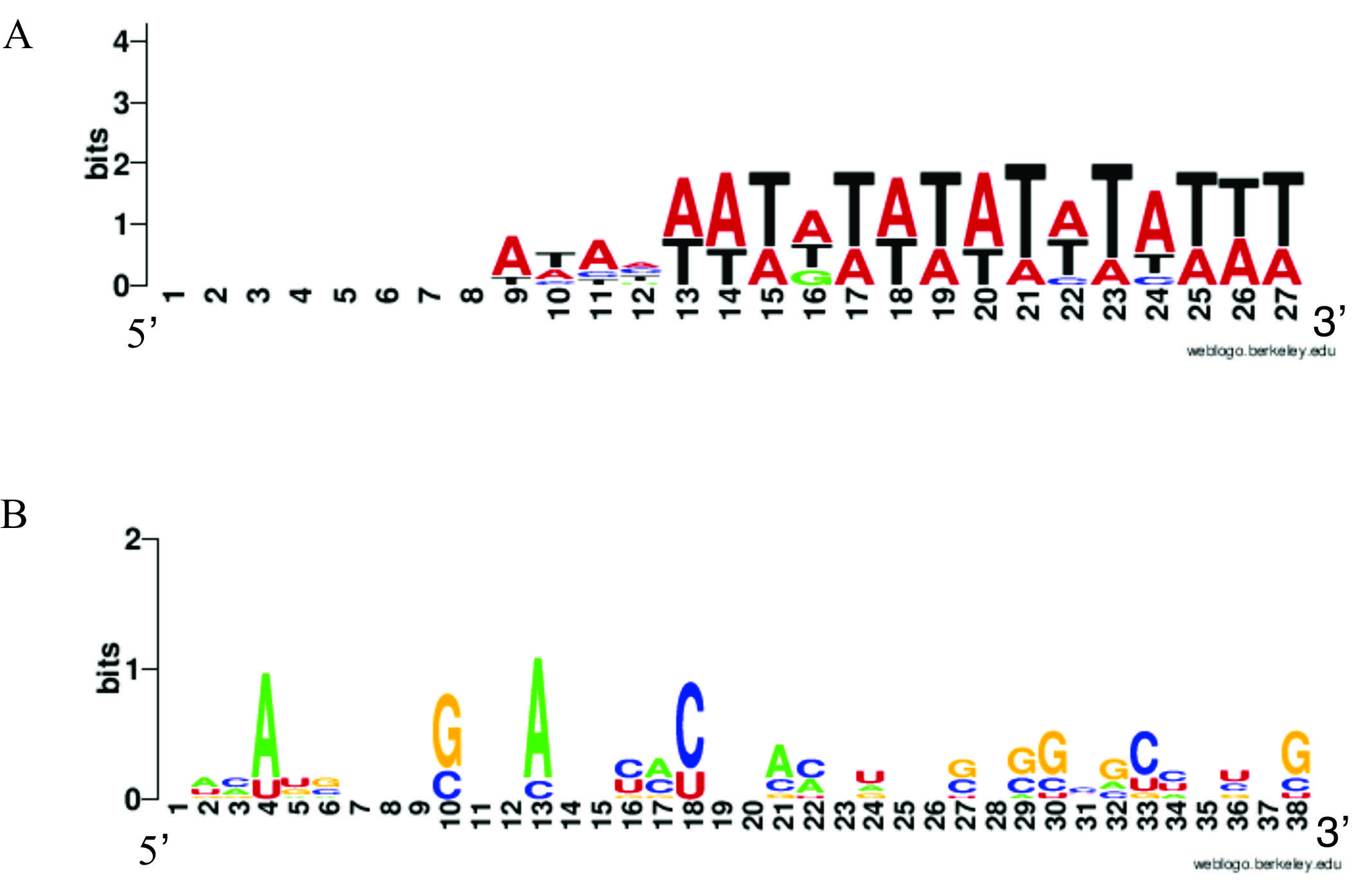

Supplement: Supplementary file 1 [file plants-10-00576-s001.zip › plants-1134083-revised suppl/Sup Fig 1 - logos plots.jpg]

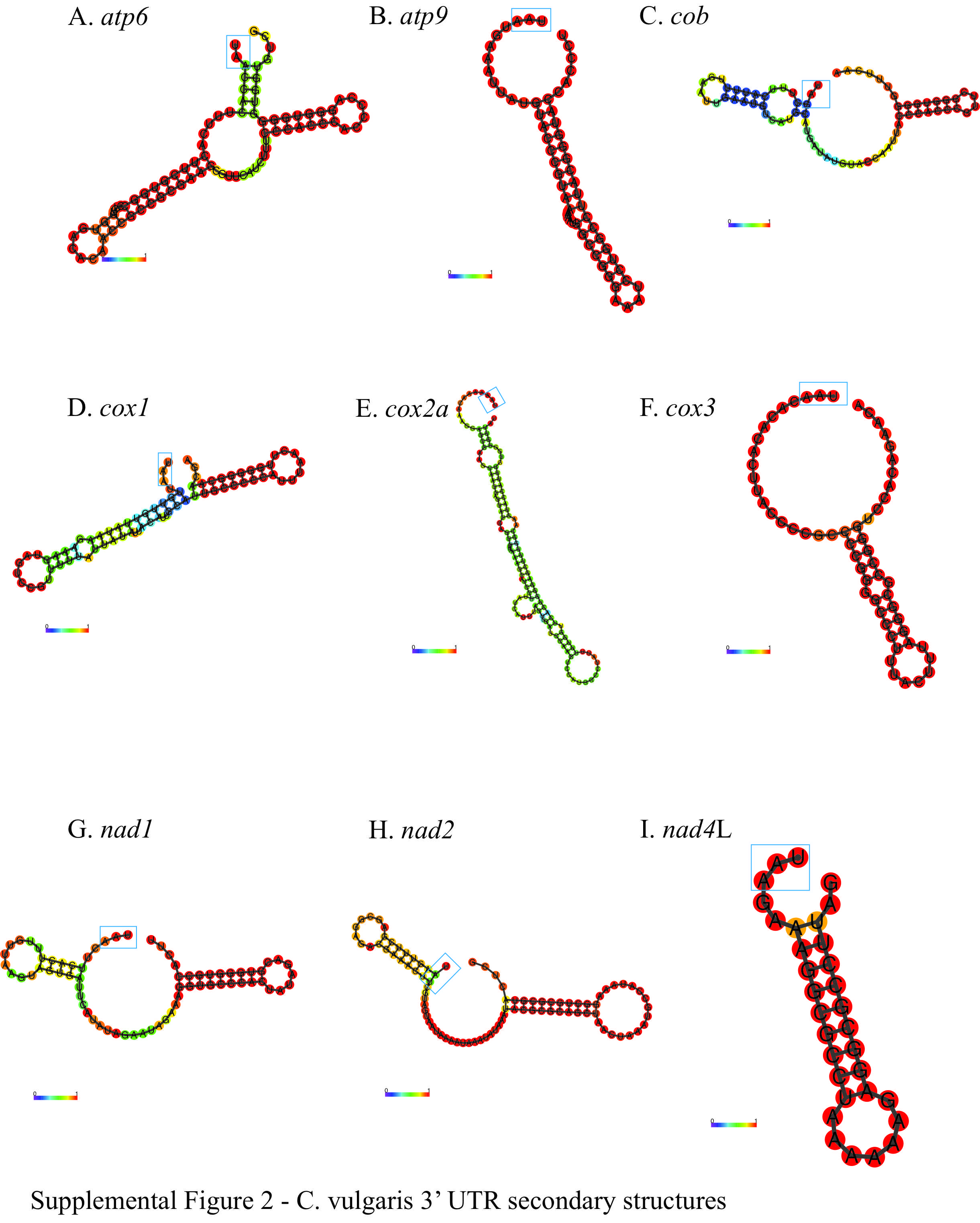

Supplement: Supplementary file 1 [file plants-10-00576-s001.zip › plants-1134083-revised suppl/Sup Figure 2 - RNA secondary structure.jpg]
